# Supplementary material for: Mitochondrial Control Region Variants Related to Breast Cancer
Source: Genes (Basel). 2022 Oct 27;13(11):1962. doi: 10.3390/genes13111962 (PMC9690046; doi:10.3390/genes13111962)
Supplement: Supplementary file 1 [file genes-13-01962-s001.zip › Table S1_Number of samples anayzed.pdf]

Number of sequences and type of sample	ID *GenBank*	# sequences (Reference)
52<br>Complete breast cancer sequences (16.5 kb)	EF660933.1, EF660934.1, EF660935.1, EF660936.1, EF660937.1, EF660938.1, EF660939.1, EF660940.1, EF660941.1, EF660942.1, EF660943.1, EF660944.1, EF660945.1, EF660946.1, EF660947.1, EF660948.1, EF660949.1, EF660950.1, EF660951.1, EF660952.1.	20<br>(Gasparre et al. 2007)
	EF114271.1, EF114273.1, EF114276.1, EF114279.1, EF114282.1, EF114285.1, EF114288.1, EF429133.1, EF429136.1, EF429139.1.	10<br>(Wang et al. 2007)
	GU592034.1, GU592035.1, GU592036.1, GU592037.1, GU592038.1, GU592039.1, GU592040.1, GU592041.1, GU592042.1, GU592043.1, GU592044.1, GU592045.1, GU592046.1, GU592047.1, GU592048.1.	15<br>(Fendt et al. 2011)
	MH550161.1, MH550162.1, MH550163.1, MH550164.1, MH550165.1.	5<br>(Hernández-de la Cruz et a. 2018)
	AB626609.1, AB626610.1.	2<br>(Imanishi et al. 2011)
38<br>Partial breast cancer sequences (less than 0.5 Kb)	HG825991.1, HG825992.1, HG825993.1, HG825994.1, HG825995.1, HG825996.1, HG825997.1, HG825998.1, HG825999.1, HG826000.1, HG826001.1, HG826002.1, HG826003.1, HG826004.1	14<br>Ghatak et al. 2014.
	DQ143184.1, DQ143185.1, DQ143186.1, DQ143187.1, DQ143188.1, DQ143189.1, DQ143190.1, DQ143191.1, DQ143192.1, DQ143193.1, DQ143194.1, DQ143195.1, DQ143196.1, DQ143197.1, DQ143198.1, DQ143199.1, DQ143200.1, DQ143201.1, DQ143202.1, DQ143203.1, DQ143204.1, DQ143205.1, DQ143206.1, DQ143207.1	24<br>Darvishi et al. 2015
34<br>Controls samples (16.5 kb)	EF114272.1, EF114275.1, EF114278.1, EF114281.1, EF114284.1, EF429135.1, EF429138.1, EF429141.1, EF114287.1.	9<br>Distant normal tissue obtained of same patients with breast cancer contralateral breast (Wang et al. 2007).
	EF114271.1, EF114274.1, EF114277.1, EF114280.1, EF114283.1, EF114286.1, EF114289.1, EF429140.1, EF429137.1, EF429134.1	10<br>Para-cancerous normal tissue (Wang et al. 2007).
	GU592019.1, GU592020.1, GU592021.1, GU592022.1, GU592023.1, GU592024.1, GU592025.1, GU592026.1, GU592027.1, GU592028.1, GU592029.1, GU592030.1, GU592031.1, GU592032.1, GU592033.1.	15<br>Laser capture microdissection obtained from biopsies and macroscopic appearance (Fendt et al. 2011).

Table S1. Origin and references of mtDNA sequences associated with breast cancer and controls used for the analysis.
