# Supplementary material for: Mitochondrial Control Region Variants Related to Breast Cancer
Source: Genes (Basel). 2022 Oct 27;13(11):1962. doi: 10.3390/genes13111962 (PMC9690046; doi:10.3390/genes13111962)
Supplement: Supplementary file 1 [file genes-13-01962-s001.zip › Table S2 Comparative macrohaplogroups distribution.pdf]

Table S2. Comparative summary of macrohaplogroups' distribution obtained from complete and partial mitochondrial sequences from patients diagnosed with breast cancer.

	**Complete mtDNA sequences (All mitochondrial chromosome)**		**Partial mtDNA sequences (Control region and hypervariable regions)**	
**Macrohaplogroup**	**Number of individual sequences**	**%**	**Number of individual sequences**	**%**
**A**	2	2.4	-	-
**B**	3	3.5	3	7.9
**C**	1	1.2	1	2.6
**D**	4	4.7	3	7.9
**E**	1	1.2	1	2.6
**F**	1	1.2	-	-
**H**	44	50.6	11	29.0
**J**	4	4.7	-	-
**K**	1	1.2	-	-
**M**	2	2.4	1	2.6
**N**	5	6.0	1	2.6
**R**	3	3.5	1	2.6
**T**	3	3.5	3	7.9
**U**	6	7.0	11	29.0
**V**	2	2.4	-	-
**X**	2	2.4	-	-
**Z**	2	2.4	2	5.3
	86	100	38	100

More than half of the complete sequences and more than a quarter of partial samples studied belong to European origin. For detailed information about haplogroups, haplotypes and subclades assigned, consult *Supplemental Table S2*.
