# Supplementary material for: Mitochondrial Control Region Variants Related to Breast Cancer
Source: Genes (Basel). 2022 Oct 27;13(11):1962. doi: 10.3390/genes13111962 (PMC9690046; doi:10.3390/genes13111962)
Supplement: Supplementary file 1 [file genes-13-01962-s001.zip › Table S3_Ghost polymorphisms.pdf]

Table S3. Comparative summary of the distribution of possible phantom polymorphisms in complete mtDNA sequences with breast cancer diagnosis.

**Position**	**Amount**	**Soares Score**
3106d	47	0
3107C	47	0
310.1C	39	0
514d	9	0
515d	9	0
398d	8	0
248d	6	0
430A	4	0
49T	4	0
16011d	4	0
8279d	3	0
8272d	3	0
8271d	3	0
8274d	3	0
8273d	3	0
8276d	3	0
8275d	3	0
8278d	3	0
8277d	3	0
16184A	3	1
12562G	2	0
16032.1G	2	0
16258d	2	0
16189d	2	0
16422A	2	0
397T	2	0
37G	2	0
16012d	2	0
574C	2	2
16353T	2	1
16390R	2	0
513.1CA	2	0
284.1C	2	0
285.1A	2	0

For more detailed information like *Genbank* ID number and haplogroups assigned, consult *Supplemental Table S4*.
