# Supplementary material for: Mitochondrial Control Region Variants Related to Breast Cancer
Source: Genes (Basel). 2022 Oct 27;13(11):1962. doi: 10.3390/genes13111962 (PMC9690046; doi:10.3390/genes13111962)
Supplement: Supplementary file 1 [file genes-13-01962-s001.zip › Table S4 Summary of the heteroplasmic in mtDNA.pdf]

Table S4. Summary of the heteroplasmic in mtDNA sequences by position in cases diagnosed with breast cancer.

Complete sequences heteroplasmy

**Position**	***Genbank* ID sequence number**	**Heteroplasmic polymorphism**
1632	GU592046.1	Y
2145	GU592041.1	R
2275	EF114285.1	Y
2998	GU592041.1	Y
5703	GU592036.1	R
7379	GU592035.1	R
7814	EF660946.1	R
7818	EF660934.1	R
8601	EF114276.1	R
9119	EF660937.1	R
9387	EF660950.1	R
12803	GU592045.1	S
13466	EF660947.1	R
15341	GU592040.1	Y
15623	GU592038.1	R
15843	EF660946.1	Y
16106	EF660946.1	M
16182	GU592037.1	M
16189	GU592040.1	M
16189	GU592046.1	M
16390	GU592042.1	R

Control sequence heteroplasmy

**Position**	***Genbank* ID sequence number**	**Heteroplasmic polymorphism**
152	GU592032.1	Y
215	GU592030.1	R
8601	GU592047.1	R
5192	GU592031.1	R
5390	GU592031.1	R
15078	GU592032.1	R
15623	GU592023.1	R
16391	GU592023.1	R

The sequence GU592023.1 was identified as a heteroplasmic position in 16401, while GU592042.1and GU592046.1 was identified in position 16400. The significance of heteroplasmic polymorphism is code following IUPAC single letter codes: symbol *R* name *Purine* and remarks *A* or *G*; symbol *Y* Name *Pyrimidine* and remarks *C* or *T*; symbol *S* name *Strong* and remarks *C* or *G*; and symbol *M* are used for *A* or *C*.
