# Supplementary material for: Mitochondrial Control Region Variants Related to Breast Cancer
Source: Genes (Basel). 2022 Oct 27;13(11):1962. doi: 10.3390/genes13111962 (PMC9690046; doi:10.3390/genes13111962)
Supplement: Supplementary file 1 [file genes-13-01962-s001.zip › Table S5 Summary of the heteroplasmic polymorphisms detected.pdf]

Table S5. Summary of the heteroplasmic polymorphisms detected by sequence and not related with haplotyping in the mtDNA sequences of cases diagnosed with breast cancer and controls.

Breast cancer mitocondrial heteroplasmy

***Genebank* ID number sequence**	**Position in mtDNA sequence**	**Type of Heteroplasmy**	**Haplogroup assigned**
GU592046.1	1632	Y	M35b (M35b2)
	16189	M	
GU592041.1	2145	R	K1a (K1a1b1c)
	2998	Y	
EF660946.1	7814	R	V2 (V2)
	15843	Y	
	16106	M	
GU592040.1	15341	Y	H65a (H65a)
	16189	M	
EF114285.1	2275	Y	B5a (B5a2a1a)
GU592036.1	5703	R	R0a (R0a1a3)
GU592035.1	7379	R	H1a (H1ay)
EF660934.1	7818	R	H1h (H1h1)
EF114276.1	8601	R	D4j (D4j3a)
EF660937.1	9119	R	N1a (N1a3a3)
EF660950.1	9387	R	U5a (U5a2c2)
GU592045.1	12803	S	HV11a (HV11a)
EF660947.1	13466	R	H7a (H7a1a)
GU592038.1	15623	R	H11a (H11a5)
GU592037.1	16182	M	H1g (H1g1)
GU592042.1	16390	R	H3q (H3q1)
GU592047.1	8601	R	J1c (J1c11a)

Control sequences

Genebank ID number sequence	Position in mtDNA sequence	Type of Heteroplasmy	Haplogroup assigned	Source
GU592031.1	5192<br>5390	R<br>R	M35b (M35b2)	LCM normal cells control sample of GU592046.1
GU592023.1	15623<br>16391	R<br>R	H11a (H11a5)	LCM normal cells control sample of GU592038.1
GU592032.1	152<br>15078	Y<br>R	J1c (J1c11a)	LCM normal cells control sample of GU592047.1
GU592030.1	215	R	HV11 (HV11a)	LCM normal cells control sample of GU592045.1

The meaning of heteroplasmic polymorphism are code following IUPAC single letter codes: symbol *R* name *Purine* and remarks *A* or *G*; symbol *Y* Name *Pyrimidine* and remarks *C* or *T*; symbol *S* name *Strong* and remarks *C* or *G*; and symbol *M* are used for *A* or *C*. LCM: laser capture microdissection.
