# Supplementary figures and images for: Mitochondrial Control Region Variants Related to Breast Cancer
Source: Genes (Basel). 2022 Oct 27;13(11):1962. doi: 10.3390/genes13111962 (PMC9690046; doi:10.3390/genes13111962)

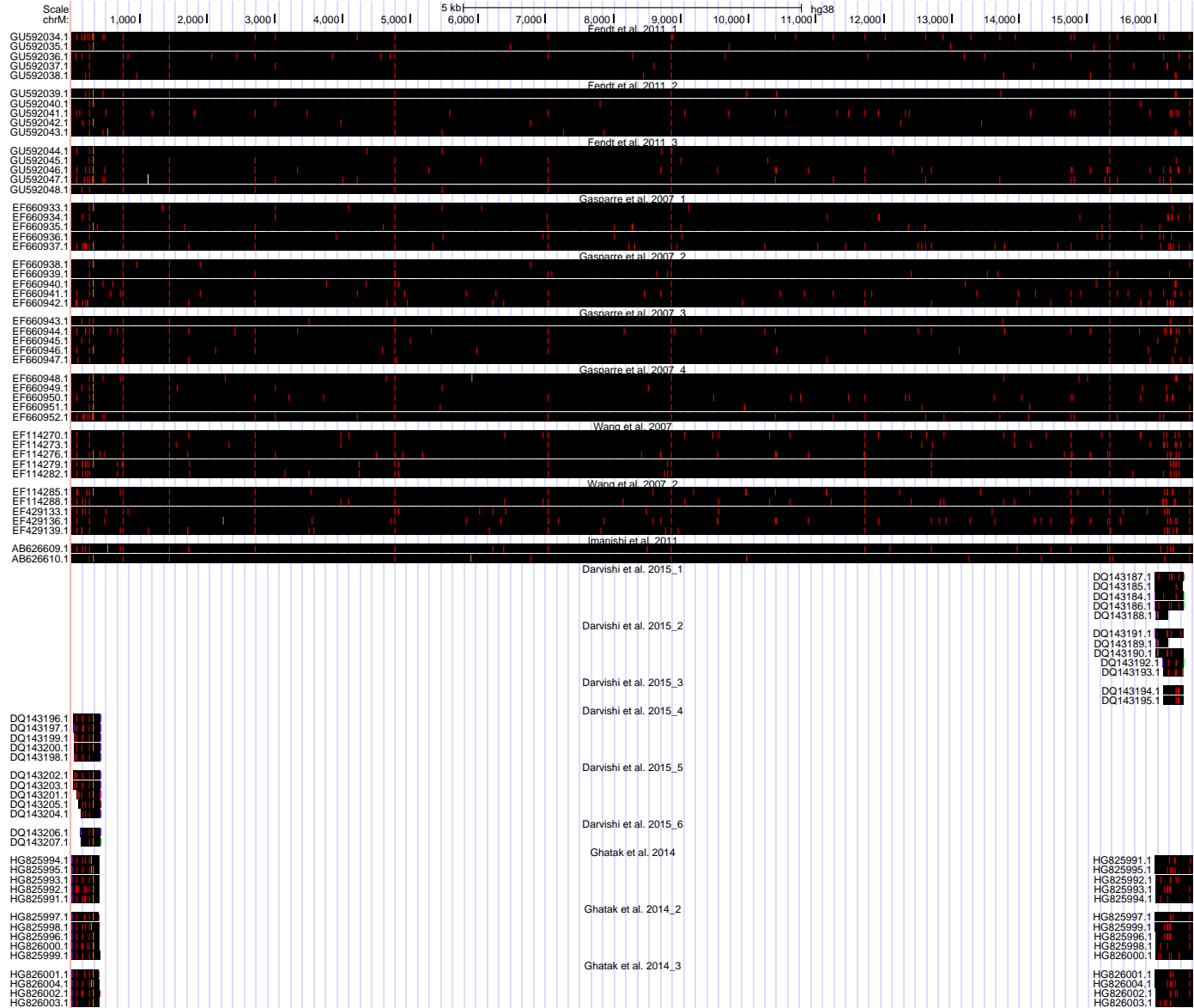

Supplement: Supplementary file 1 [file genes-13-01962-s001.zip › Baptista Rosas et al. 2022 Figure S2.pdf]

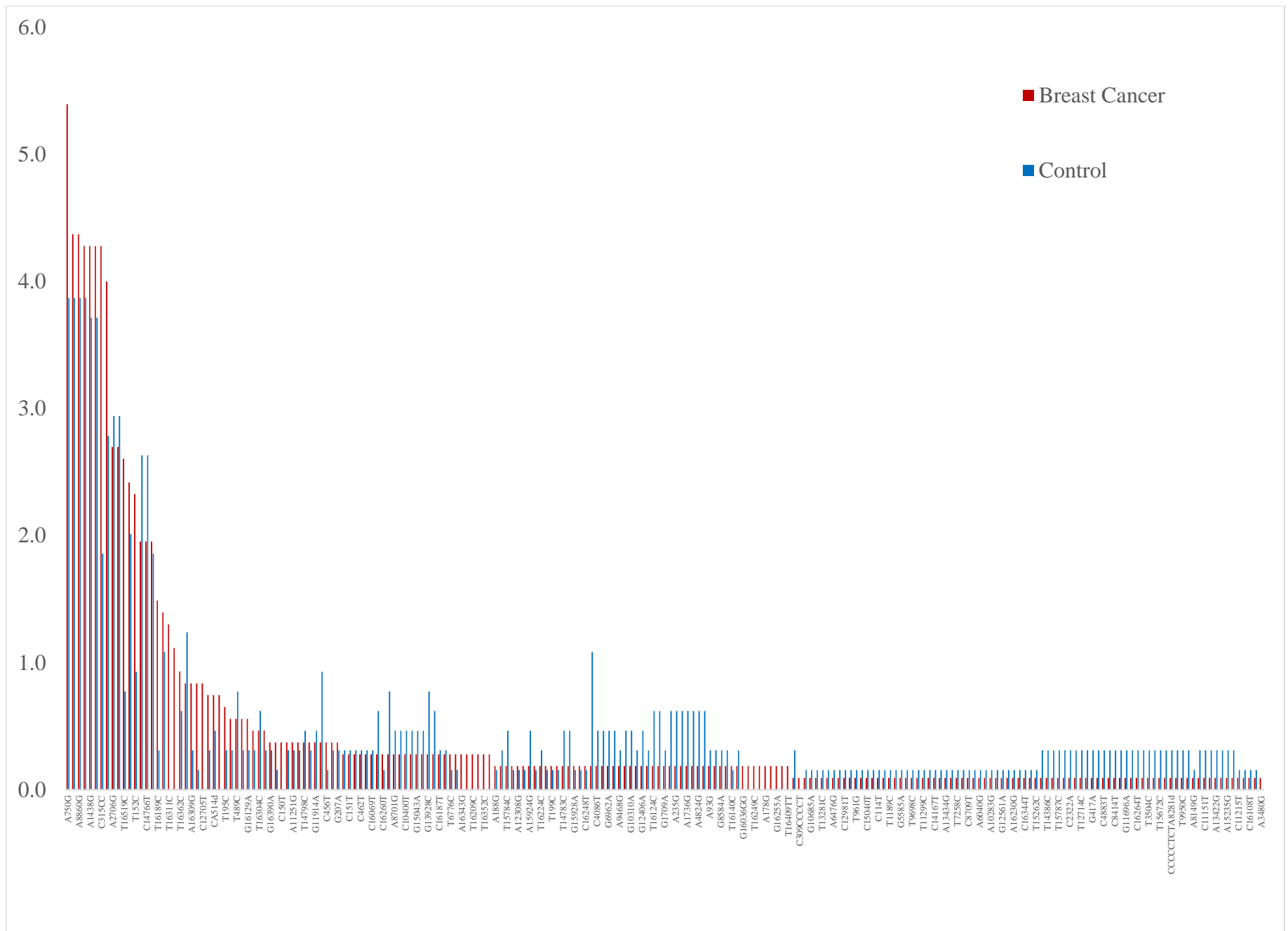

Supplement: Supplementary file 1 [file genes-13-01962-s001.zip › Baptista Rosas et al. 2022 Figure S3.pdf]

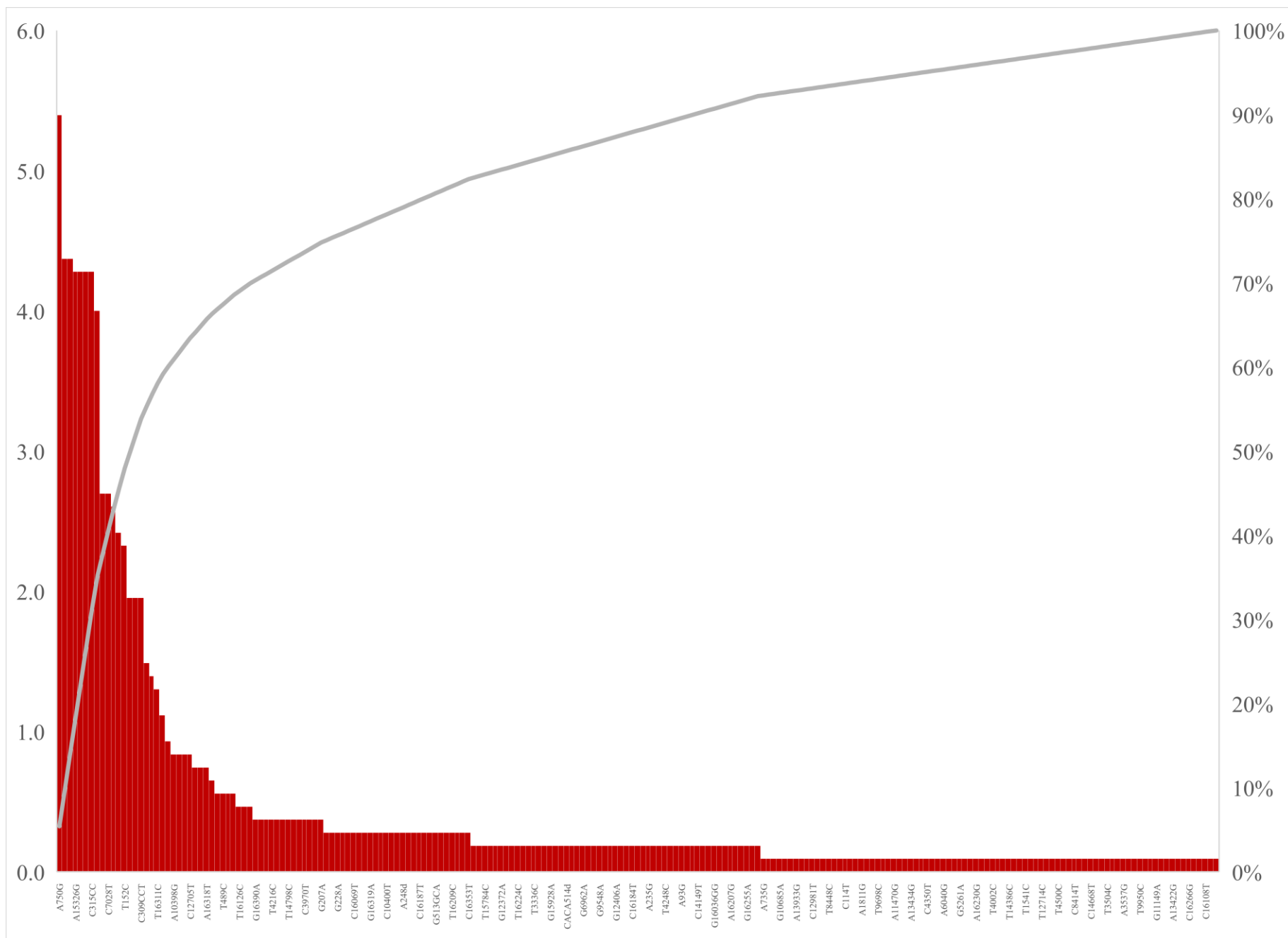

Supplement: Supplementary file 1 [file genes-13-01962-s001.zip › Baptista Rosas et al. 2022 Figure S4.pdf]

■ Breast Cancer ■ Control

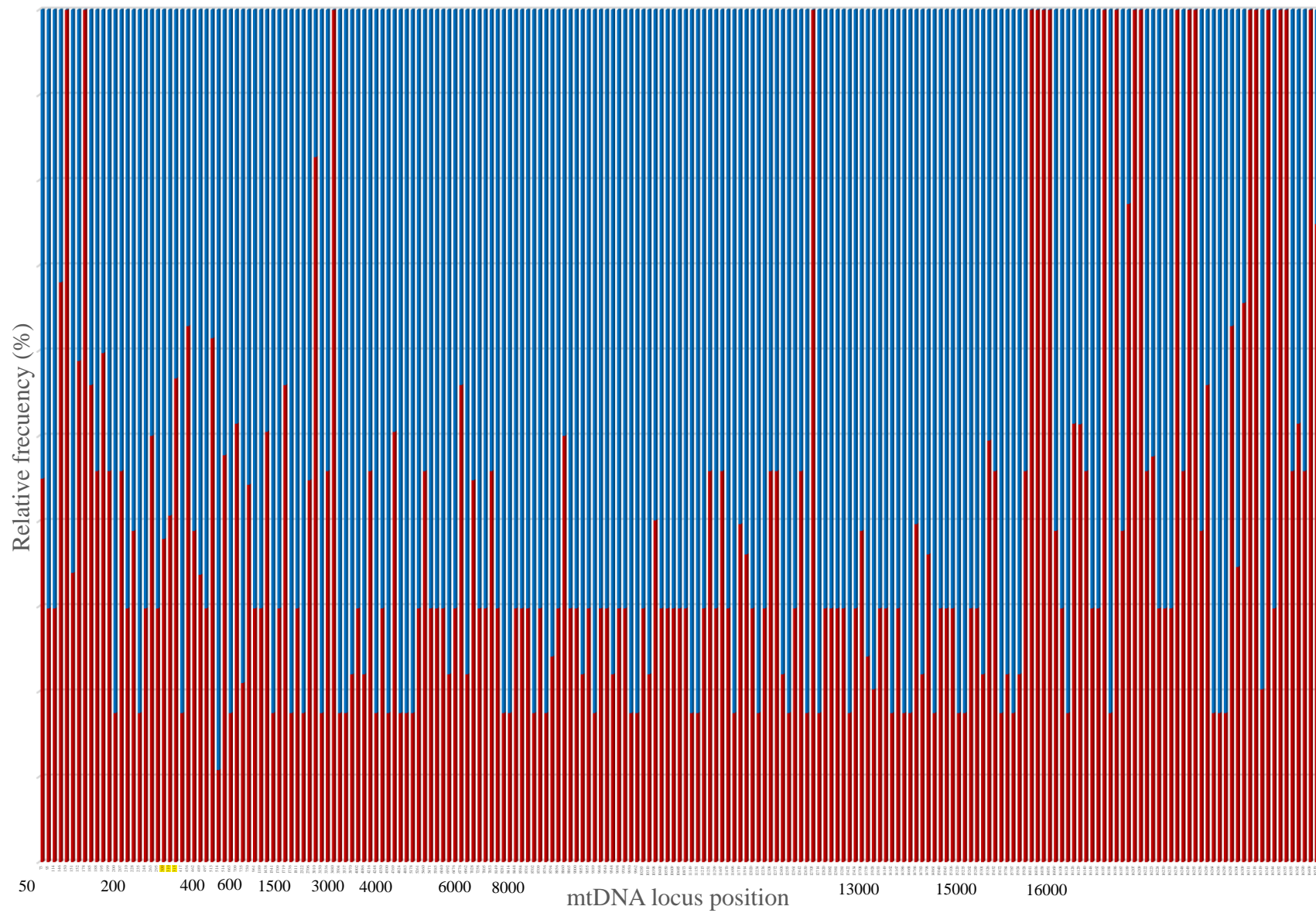

Supplement: Supplementary file 1 [file genes-13-01962-s001.zip › Baptista Rosas et al. 2022 Figure S5.pdf]

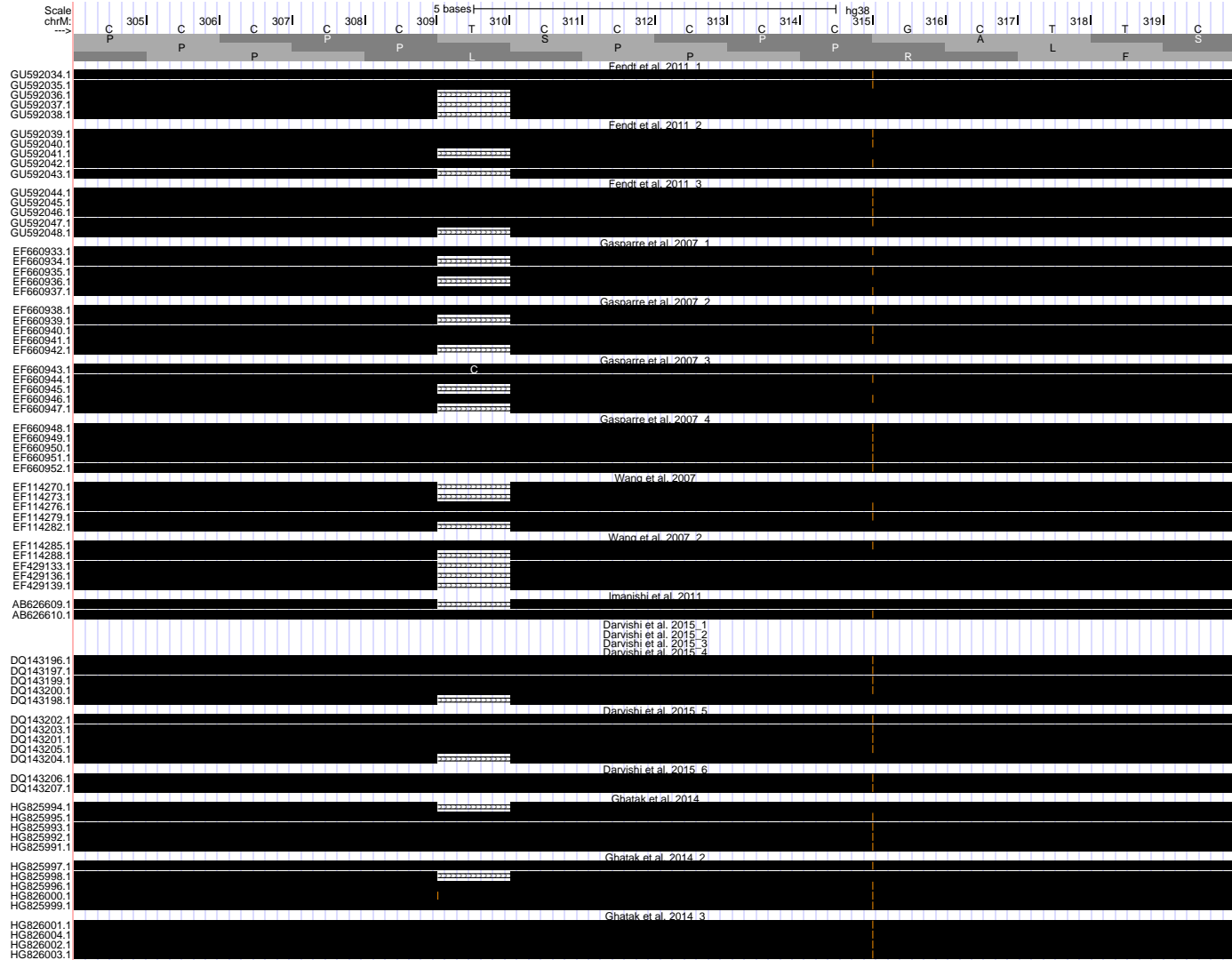

Supplement: Supplementary file 1 [file genes-13-01962-s001.zip › Baptista Rosas et al. 2022 Figure S6.pdf]

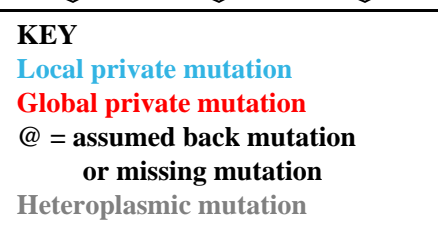

Supplement: Supplementary file 1 [file genes-13-01962-s001.zip › Baptista Rosas et al. 2022 Figure S1.pdf]
